# Genetic analysis and outcomes of Omani children with steroid‐resistant nephrotic syndrome

**DOI:** 10.1002/mgg3.2201

**Published:** 2023-05-19

**Authors:** Mohamed S. Al Riyami, Intisar Al Alawi, Badria Al Gaithi, Anisa Al Maskari, Naifain Al Kalbani, Nadia Al Hashmi, Aisha Al Balushi, Maryam Al Shahi, Suliman Al Saidi, Muna Al Bimani, Fahad Al Hatali, Holly Mabillard, John A. Sayer

**Affiliations:** ^1^ Pediatric Nephrology Unit, Department of Child Healthy Royal Hospital Muscat Oman; ^2^ Translational and Clinical Research Institute, Faculty of Medical Science Newcastle University Newcastle upon Tyne UK; ^3^ National Genetic Center, Ministry of Health Muscat Oman; ^4^ Pediatric Metabolic and Genetic Disorder Unit Royal Hospital Muscat Oman; ^5^ Pediatric Clinical Genetic Unit, Royal Hospital, Department of Child Health Royal Hospital Muscat Oman; ^6^ Renal Services The Newcastle upon Tyne Hospitals NHS Foundation Trust Newcastle upon Tyne UK; ^7^ Newcastle Biomedical Research Center, NIHR Newcastle upon Tyne UK

**Keywords:** edema, hyperlipidemia, hypoalbuminemia, kidney failure, proteinuria, steroid‐resistant nephrotic syndrome

## Abstract

**Background:**

Nephrotic syndrome (NS) is one of the most common kidney disorders seen by pediatric nephrologists and is defined by the presence of heavy proteinuria (>3.5 g/24 h), hypoalbuminemia (<3.5 g/dL), edema, and hyperlipidemia. Most children with NS are steroid‐responsive and have a good prognosis following treatment with prednisolone. However, 10%–20% of them have steroid‐resistant nephrotic syndrome (SRNS) and fail to respond to treatment. A significant proportion of these children progress to kidney failure.

**Methods:**

This retrospective study aimed to determine the underlying genetic causes of SRNS among Omani children below 13 years old, over a 15‐year period and included 77 children from 50 different families. We used targeted Sanger sequencing combined with next‐generation sequencing approaches to perform molecular diagnostics.

**Results:**

We found a high rate of underlying genetic causes of SRNS in 61 (79.2%) children with pathogenic variants in the associated genes. Most of these genetically solved SRNS patients were born to consanguineous parents and variants were in the homozygous state. Pathogenic variants in *NPHS2* were the most common cause of SRNS in our study seen in 37 (48.05%) cases. Pathogenic variants in *NPHS1* were also seen in 16 cases, especially in infants with congenital nephrotic syndrome (CNS). Other genetic causes identified included pathogenic variants in *LAMB2*, *PLCE1*, *MYO1E*, and *NUP93*.

**Conclusion:**

*NPHS2* and *NPHS1* genetic variants were the most common inherited causes of SRNS in Omani children. However, patients with variants in several other SRNS causative genes were also identified. We recommend screening for all genes responsible for SRNS in all children who present with this phenotype, which will assist in clinical management decisions and genetic counseling for the affected families.

## Graphical abstract

Nephrotic syndrome is one of the most common kidney disorders seen by pediatric nephrologists. This study in 77 Omani children determined the underlying genetic causes of steroid‐resistant nephrotic syndrome (SRNS). Pathogenic variants in NPHS2 were the most common cause of SRNS in our study seen in almost half of all cases. We recommend screening for all genes responsible for SRNS in all children who present with this phenotype, which will assist in clinical management decisions and genetic counseling for the affected families.

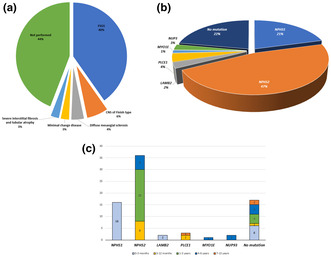

## 1 INTRODUCTION

Idiopathic nephrotic syndrome (INS) is one of the most common kidney disorders seen in the pediatric clinic with an estimated incidence of approximately 2–6.5 per 100,000 children per year, depending on ethnicities and regions (McKinney et al., 2001). The presence of heavy proteinuria (protein excretion >3.5 g/24 h), hypoalbuminemia (<3.5 g/dL), edema, and hyperlipidemia are the main clinical manifestations (Eddy & Symons, 2003). Most children with NS are steroid‐responsive and have a good prognosis, while 10%–20% of them exhibit steroid resistance and do not achieve complete remission within 4–6 weeks of glucocorticoid treatment (Trautmann et al., 2020), hence are labeled as steroid‐resistant nephrotic syndrome (SRNS). Outcomes for children with non‐genetic SRNS are generally good with around two‐thirds of patients achieving complete remission (Trautmann et al., 2023). For the genetic subgroup of SRNS, complete remission is more uncommon. Around 20% of genetic SRNS patients have chronic kidney disease (CKD), progress to kidney failure (KF) (Trautmann et al., 2023) and no response to immunosuppression in the first year is a predictor of poor kidney function outcomes (Ying et al., 2021). The most common histological diagnoses in children with NS include minimal change disease (MCD), focal segmental glomerulosclerosis (FSGS), membranoproliferative glomerulonephritis and membranous glomerulonephritis (Churg et al., 1970). A histological picture of FSGS is commonly seen in children with SRNS (Churg et al., 1970; McCarthy & Saleem, 2011).

The etiology of idiopathic SRNS in children is either due to a single gene disorder leading to abnormal expression of a podocyte specific protein (Machuca et al., 2009) or due to immune dysregulation which probably leads to production of circulating glomerular permeability factors (Machuca et al., 2009; Shalhoub, 1974). Several genetic causes are known to be involved in the pathogenesis of SRNS (OMIM PS256300) (Ha, 2017; Koziell et al., 2002). Mutations in *NPHS1* (OMIM 602716), *NPHS2* (OMIM 604766), *WT1* (OMIM 256370), *LAMB2* (OMIM 614199) and *PLCE1* (OMIM 610725) are the most common causes of genetic forms of NS in children (Ha, 2017). These genes encoded different proteins contributing to the glomerular filtration barrier and defects lead to proteinuria and deterioration of kidney function (Wiggins, 2007) and eventually development of KF (Ha, 2017; Koziell et al., 2002). Recent advances in next‐generation sequencing (NGS) have enabled the identification of more than 50 podocyte‐related genes associated with different monogenic forms of NS (Bierzynska et al., 2017; Preston et al., 2019) including *MYO1E* (OMIM 614131) and *NUP93* (OMIM 616892).

For children with SRNS due to a genetic cause, a molecular genetic diagnosis is essential for decision‐making related to treatment and predicting prognosis (Ha, 2017; Shalhoub, 1974). It enables clinicians to start appropriate management and treatment. In addition, patients with disease‐causing variants in some of the genes encoding enzymes of the coenzyme Q10 (CoQ10) pathway (e.g., *COQ2* (OMIM 607426) and *COQ6* (OMIM 614650)) can be treated using supplementation with CoQ10 (Preston et al., 2019). Furthermore, identification of disease‐causing mutations can facilitate the process of kidney donor selection and the prediction of post‐transplant recurrence of NS (Morello et al., 2023).

In Oman, inherited monogenic kidney diseases are relatively common, leading to a significant healthcare burden (Al Alawi, Al Salmi, Al Mawali, Al Maimani, & Sayer, 2017; Al Alawi, Al Salmi, Al Mawali, & Sayer, 2017). Molecular genetic studies of patients with inherited cystic kidney disease and renal ciliopathies from this population have previously been reported (Al Alawi et al., 2019, 2020, 2021). This present study describes, for the first time, the demographic characteristics, clinical features, and genetic findings of children with SRNS who were seen in the pediatric nephrology department at the Royal Hospital, Oman. The identification of the genetic components of these children with SRNS is essential given the tribal structure of the Omani population and the high rate of consanguineous marriages. This report gives insight into the type and frequency of genetic mutations detected in Omani children with SRNS and is anticipated to support pediatricians and pediatric nephrologists in management decisions and genetic counseling.

## 2 METHODS

### 2.1 Ethical compliance

This study was ethically approved by the Royal Hospital Research Ethical Committee, Ministry of Health (MOH/CSR/21/24412). For genetic studies of patients, written informed consent was provided by the patients' families. Genomic DNA was isolated from whole blood of patients and the available family members at the time of presentation using Hamilton's Microlab® STAR™, according to the manufacturer protocol.

### 2.2 Patient identification and recruitment

This retrospective study involved reviewing 77 pediatric patients (≤13 years old) from 50 Omani families diagnosed with primary SRNS at Royal Hospital, Oman, over a period of 15 years (2005 to 2020). The hospital electronic medical records of all 77 patients were retrospectively reviewed. The following variables were collected: age at onset of NS, gender, parental consanguinity, family history of disease (including previous genetic diagnosis in a sibling), and initial symptoms. Data on kidney function at presentation and last follow‐up, serum albumin, kidney biopsy findings, immunosuppressive treatment, renal replacement therapy including dialysis/transplant, and outcome were also collected.

This study was ethically approved by the Royal Hospital Research Ethical Committee, Ministry of Health (MOH/CSR/21/24412). For genetic studies of patients, written informed consent was provided by the patients' families. Genomic DNA was isolated from whole blood of patients and the available family members including other affected siblings and parents at the time of presentation using Hamilton's Microlab® STAR™, according to the manufacturer protocol. Genetic analysis, described below, was performed as a clinical service on incident cases.

Patients were diagnosed with SRNS based on the following criteria: failure to respond (absence of complete remission, defined as resolution of proteinuria) to prednisolone 60 mg/m^2^/day for 4–6 weeks with or without three doses of intravenous methylprednisolone. Patients were diagnosed with congenital nephrotic syndrome (CNS) if symptoms presented on the first 3 months of life, whereas they were classified with infantile nephrotic syndrome if presented between 3 and 12 months of age. Patients presenting with NS between 1 and 13 years of age and not responding to steroids were labeled as childhood SRNS. Patient were diagnosed with multidrug resistance based on absence of complete remission after 12 months of treatment with steroid and another two immunosuppressive medications. Prolonged high‐dose steroid was not given to patients presenting with SRNS in the context of a sibling with a known genetic diagnosis of SRNS or if there was established KF.

High blood pressure was defined according to the 4th report on diagnosis, evaluation, and treatment of high blood pressure in children and adolescents (Hogg et al., 2003).

Glomerular filtration rate (GFR) was calculated from the Schwartz equation; normal GFR was defined if GFR ≥90 mL/min/1.73 m^2^. CKD was staged according to published CKD classification (Eckardt et al., 2009; Hogg et al., 2003).

Regarding the treatment of SRNS children in our center, the following were usually practiced:

- The majority of patients with CNS were managed by daily intravenous albumin infusion in addition to receiving anti‐proteinuric measures using ACE inhibitor and indomethacin, nutrition support and management of complications including infection, anemia, and hypothyroidism.
- Patients diagnosed with childhood SRNS were started on anti‐proteinuric measures using ACE inhibitors.
- In cases where there was an absence of genetic testing or failure to detect disease‐causing variants in *NPHS1* or *NPHS2*, children were treated with calcineurin inhibitor cyclosporine or tacrolimus for a minimum of 6 months, and some of them received mycophenolate and or rituximab before being labeled as multidrug resistance NS.

### 2.3 Molecular testing of *NPHS1* and *NPHS2*

Molecular analysis of *NPHS1* and *NPHS2* genes was performed using targeted Sanger sequencing of patient DNA at the National Genetic Centre, Royal Hospital, Oman. All coding exons, adjacent exon‐intron boundaries and up to 700 bp of the 5′UTR end from both *NPHS1* and *NPHS2* genes were amplified using polymerase chain reaction (PCR). Primer3 was utilized to design primer sequences (http://primer3.ut.ee/), which are available upon request. PCR amplification was performed using Ampli*Taq* Gold PCR master mix (Qiagen) kit, as per the manufacturer instructions. The amplified amplicons were verified on 1% gel and purified using ExoSAP‐IT PCR clean‐up reagent (Applied Biosystems). Bi‐directional fluorescence‐based sequencing was performed on an ABI 3730 XL sequencer using BigDye Terminator V3.1 Cycle Sequencing kit (Applied Biosystems). The obtained sequences were assembled and aligned compared with a reference sequence using the SequencePilot 4.2.2 software (JSI Medical Systems GmbH).

### 2.4 Genetic variant detection and annotation

Genetic variants were assessed as clinically significant by using public databases including Human Gene mutation database (HGMD), LOVD and variants experimentally evidenced in the literature to be associated with disease phenotype. Variants commonly seen in healthy population with frequency ≥1% were excluded from analysis using databases 1000 Genomes project, Exome Aggregation Consortium (ExAC), Genome Aggregation Database (gnomAD), and NHLBI ESP. In addition, ACMG variant classification was performed using Varsome. GenBank Reference Sequences used were *NPHS1* (NM_004646.4); *NPHS2* (NM_014625.4); *LAMB2* (NM_002292.4); *MYO1E* (NM_004998.4); *PLCE1* (NM_016341.4); and *NUP93* (NM_014669.5).

### 2.5 Whole exome sequencing

Ten patients, in whom no genetic mutations in *NPHS1* and *NPHS2* were detected, underwent further genetic testing using whole exome sequencing (WES) via CENTOGENE, Germany, or the Translational and Clinical Research Institute, Newcastle University, UK.

### 2.6 *In silico* modeling of *PLCE1* and *NUP93* alleles

Alphafold sequence AF‐Q9P212‐F1 was imported to PyMOL and labeled according to available UniProtKB data. PyMOL Mutation Wizard was used to model the most likely structural impact of c4301G>T, p.(Arg1434Leu) in the 1‐phosphatidylinositol 4,5‐bisphosphate phosphodiesterase epsilon‐1 protein, encoded from the *PLCE1* gene on position 23.33 the q arm of chromosome 10 and has 39 exons. Due to the availability of human protein crystal structure informed by nuclear magnetic resonance and X‐ray crystallography, the mutational region of interest is predicted with >90% accuracy and the mutation effects are predicted with 93.6% accuracy.

Alphafold sequence AF‐Q8N1F7‐F1 was imported to PyMOL and labeled according to available UniProtKB data. PyMOL Mutation Wizard was used to model the most likely structural impact of c.1319T>C, p.(Phe440Ser) in the Nucleoporin 93 protein, encoded from the NUP93 gene on chromosome 16q13. Due to the availability of human protein crystal structure informed by electron microscopy, the mutational region of interest is predicted with >90% accuracy. The most likely rotamer has been illustrated above with mutation effects predicted with 41.9% accuracy.

## 3 RESULTS

### 3.1 Patients characteristics

In total, 77 children were included in this study from different regions of Oman, who were followed by the pediatric nephrology unit at Royal hospital, Oman. It comprised 41 females and 36 males from 50 different families. The mean age at disease onset was 17.5 months (range 0–120 months). Consanguineous marriages were reported in 58 (75%) of the total cohort and in 48 patients who had a positive family history of SRNS. Of these 77 patients, 24 (31.1%) were diagnosed with CNS, 9 (11.7%) were diagnosed with INS and the remaining 44 (57.1%) were diagnosed with childhood SRNS.

All children had nephrotic range proteinuria. Haematuria at presentation was reported in 60 (77.9%) of our patients. CKD at disease onset was seen in 23 (29.9%) patients. Hypothyroidism and hypertension were reported in 47 (61.0%) and 21 (28.0%), respectively. Mean eGFR at disease onset was 124 mL/min/1.73 m^2^ while mean eGFR at last follow‐up was 53 mL/min/1.73 m^2^. Mean serum albumin at disease onset and last follow‐up were 12.3 g/L and 26 g/L, respectively.

**FIGURE 1:**
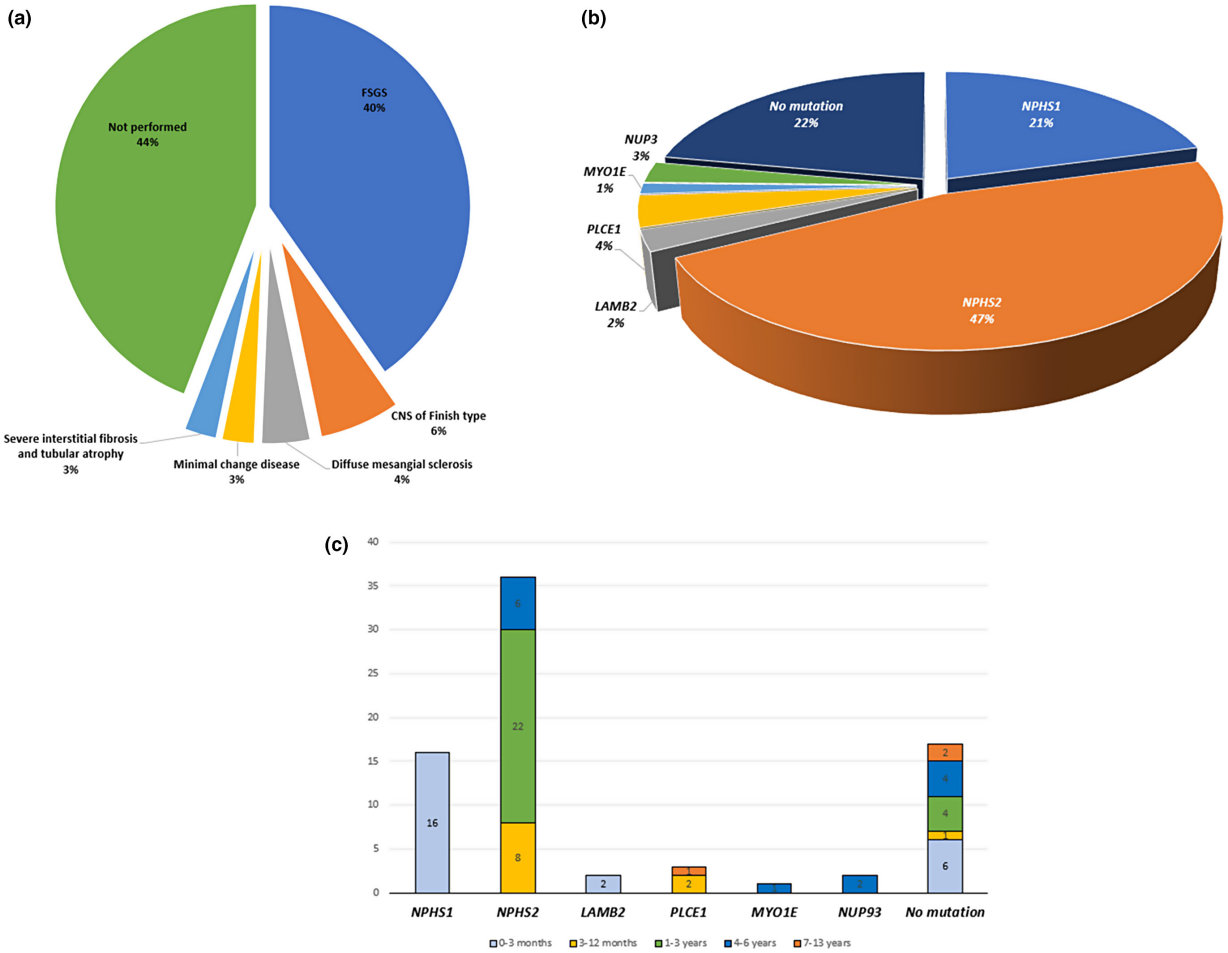
Summary of genetic and clinical studies of our steroid resistance nephrotic syndrome (SRNS) cohort. (a) Distribution of genetic findings among different age groups of patients. Histograms indicate the number of patients with causative gene detected per age group. (b) Gene‐specific distribution of identified disease‐causing variants and without genetic causes “no mutation”. (c) Histopathological analysis of patients with SRNS. FSGS; Focal segmental glomerulosclerosis. FT NS: Finish type nephrotic syndrome. DMS: diffuse mesangial sclerosis. IFTA: interstitial fibrosis and tubular atrophy.

Before 2014, most patients who had SRNS underwent a diagnostic kidney biopsy, but subsequently, biopsy rates declined as genetic testing for SRNS was introduced. After 2014, only patients who were negative for disease‐causing variants in *NPHS1* and *NPHS2* proceeded to kidney biopsy. In this cohort, a kidney biopsy was performed in 43 (55.8%) children. The most common histopathological diagnosis was FSGS (*n* = 31; 40.4%), followed by CNS of Finnish type (*n* = 5; 6.5%), diffuse mesangial sclerosis (3; 3.9%), MCD (2; 2.6%), and severe interstitial fibrosis and tubular atrophy (2; 2.6%) (Figure 1a).

Disease‐causing genetic variants were identified in 60 (77.9%) children using a combination of direct Sanger sequencing of *NPHS1* and *NPHS2* (52 patients) and WES (8 patients) (Table 1 and Figure 1b). In these 60 patients, 46 patients had homozygous disease‐causing variants, consistent with the high rates of consanguinity.

**TABLE 1:** Demographic data, phenotypic presentation and genetic analysis of the Omani SRNS patient cohort.

Family no.	Sex	Consanguinity	Age at diagnosis	Phenotype	Renal biopsy	Gene	Nucleotide change	Zygosity	Amino acid change and ACMG criteria	Patient and kidney outcomes
1a	Male	No	1 month	CNS	Not done	*NPHS1*	c.514_516delACC; c.2501T>A	Compound heterozygous	p.(Thr172del) (Pathogenic); p.(Val834Asp) (Likely Pathogenic)	Deceased
1b	Male	No	1 month	CNS	Not done	*NPHS1*	c.514_516delACC; c.2501T>A	Compound heterozygous	p.(Thr172del) (Pathogenic); p.(Val834Asp) (Likely Pathogenic)	Deceased
2	Female	Yes	1 month	CNS	FT	*NPHS1*	c.515_517 delCCA; c.1379G>A	Compound heterozygous	p.(Thr172del) (Pathogenic); p.(Arg460Gln) (Pathogenic)	KT
3	Female	Yes	1 month	CNS	Not done	*NPHS1*	c.515_517delCCA	Homozygous	p.(Thr172del) (Pathogenic)	Deceased
4a	Female	Yes	2 month	CNS	Not done	*NPHS1*	c.515_517delCCA	Homozygous	p.(Thr172del) (Pathogenic)	PD
4b	Female	Yes	1 month	CNS	FT	*NPHS1*	c.515_517delCCA	Homzygous	p.(Thr172del) (Pathogenic)	Deceased
4c	Male	Yes	1 year	INS	Not done	*NPHS1*	c.515_517delCCA	Homozygous	p.(Thr172del) (Pathogenic)	PD
5	Female	Yes	1 month	CNS	Not done	*NPHS1*	c.515_517delCCA	Homozygous	p.(Thr172del) (Pathogenic)	Deceased
6	Female	Yes	1 month	CNS	Not done	*NPHS1*	c.614_621 delCACCCCGGinsTT	Homozygous	p.(Thr205_Arg207delinsIle (Pathogenic)	Deceased
7	Female	Yes	1 month	CNS	Not done	*NPHS1*	c.515_517delCCA; c.3478C>T	Compound heterozygous	p.(Thr172del) (Pathogenic); p.(Arg160*) (Pathogenic)	CKD
8	Female	Yes	1 month	CNS	Not done	*NPHS1*	c.515_517 delCCA	Homozygous	p.(Thr172del) (Pathogenic)	Deceased
9	Male	Yes	1 month	CNS	Not done	*NPHS1*	c.515_517 delCCA	Homozygous	p.(Thr172del) (Pathogenic)	Normal GFR
10a	Female	Yes	3 month	CNS	Not done	*NPHS1*	c.2663G>A	Homozygous	p.(Arg88Lys) (Likely Pathogenic)	Deceased
10b	Female	Yes	1 month	CNS	FT	*NPHS1*	c.2663G>A	Homozygous	p.(Arg88Lys) (Likely Pathogenic)	HD
11	Female	Yes	2.5 month	CNS	FT	*NPHS1*	c.1134G>A	Homozygous	p.(Trp378*) (Likely Pathogenic)	KT
12	Male	Yes	1 month	CNS	FT	*NPHS1*	c.515_517delCCA	Homozygous	p.(Thr172del) (Pathogenic)	PD
13	Female	Yes	4 year	SRN	Not done	*NPHS2*	c.467dup	Homozygous	p.(Leu156Phefs*11) (Pathogenic)	PD
14a	Female	Yes	2 year	SRN	FSGS	*NPHS2*	c.779T>A	Homozygous	p.(Val1260Glu) (Pathogenic)	KT
14b	Female	Yes	5 year	SRN	Not done	*NPHS2*	c.779T>A	Homozygous	p.(Val1260Glu) (Pathogenic)	KT
14c	Female	Yes	3 year	SRN	Not done	*NPHS2*	c.779T>A	Homozygous	p.(Val1260Glu) (Pathogenic)	Normal GFR
15a	Male	Yes	1 year	INS	FSGS	*NPHS2*	c.467dup	Homozygous	p.(Leu156Phefs*11) (Pathogenic)	KT
15b	Male	Yes	1 year	INS	FSGS	*NPHS2*	c.467dup	Homozygous	p.(Leu156Phefs*11) (Pathogenic)	KT
15c	Female	Yes	1 year	INS	FSGS	*NPHS2*	c.467dup	Homozygous	p.(Leu156Phefs*11) (Pathogenic)	Deceased
16a	Female	Yes	2 year	SRNS	FSGS	*NPHS2*	c.467dup/c.709G>C	Compound heterozygous	p.(Leu156Phefs*11) (Pathogenic); p.(Glu237Gln) (Uncertain Significance PM1 PP1 BS2 BP4 BP6)	HD
16b	Male	Yes	2 year	SRNS	FSGS	*NPHS2*	c.467dup/c.709G>C	Compound heterozygous	p.(Leu156Phefs*11) (Pathogenic); p.(Glu237Gln)) (Uncertain Significance PM1 PP1 BS2 BP4 BP6)	HD
16c	Male	Yes	3 year	SRNS	FSGS	*NPHS2*	c.467dup/c.709G>C	Compound heterozygous	p.(Leu156Phefs*11) (Pathogenic); p.(Glu237Gln)) (Uncertain Significance PM1 PP1 BS2 BP4 BP6)	HD
17	Male	Yes	3 year	SRNS	Not done	*NPHS2*	c.467dup	Homozygous	p.(Leu156Phefs*11) (Pathogenic)	PD
18	Male	Yes	3 year	SRNS	FSGS	*NPHS2*	c.467dup	Homozygous	p.(Leu156Phefs*11) (Pathogenic)	Normal GFR
19a	Male	Yes	18 month	SRNS	Not done	*NPHS2*	c.779T>A	Homozygous	p.(Val1260Glu) (Pathogenic)	KT
19b	Female	Yes	1 year	SRNS	Not done	*NPHS2*	c.779T>A	Homozygous	p.(Val1260Glu) (Pathogenic)	PD
20a	Male	Yes	1 year	INS	FSGS	*NPHS2*	c.779T>A	Homozygous	p.(Val1260Glu) (Pathogenic)	KT
20b	Female	Yes	5 year	SRNS	Not done	*NPHS2*	c.779T>A	Homozygous	p.(Val1260Glu) (Pathogenic)	HD
20c	Female	Yes	2 year	SRNS	Not done	*NPHS2*	c.779T>A	Homozygous	p.(Val1260Glu) (Pathogenic)	Deceased
20d	Male	Yes	3 year	SRNS	MCD	*NPHS2*	c.779T>A	Homozygous	p.(Val1260Glu) (Pathogenic)	KT
20e	Male	Yes	2 year	SRNS	Not done	*NPHS2*	c.779T>A	Homozygous	p.(Val1260Glu) (Pathogenic)	HD
20f	Male	Yes	5 month	INS	Not done	*NPHS2*	c.779T>A	Homozygous	p.(Val1260Glu) (Pathogenic)	Normal GFR
21a	Female	Yes	6 year	SRNS	FSGS	*NPHS2*	c.467dup	Homozygous	p.(Leu156Phefs*11) (Pathogenic)	KT
21b	Female	Yes	2 year	SRNS	FSGS	*NPHS2*	c.467dup	Homozygous	p.(Leu156Phefs*11) (Pathogenic)	KT
21c	Female	Yes	3 year	SRNS	Not done	*NPHS2*	c.467dup	Homozygous	p.(Leu156Phefs*11) (Pathogenic)	Normal GFR
22a	Male	Yes	1.5 year	SRNS	FSGS	*NPHS2*	c.685C>T	Homozygous	p.(Arg229*) (Pathogenic)	PD
22b	Male	Yes	1 year	SRNS	FSGS	*NPHS2*	c.685C>T	Homozygous	p.(Arg229*) (Pathogenic)	PD
22c	Female	Yes	2 year	SRNS	Not done	*NPHS2*	c.685C>T	Homozygous	p.(Arg229*) (Pathogenic)	Normal GFR
23	Female	Yes	9 month	INS	FSGS	*NPHS2*	c.779T>A	Homozygous	p.(Val1260Glu) (Pathogenic)	PD
24	Male	No	5 year	SRN	FSGS	*NPHS2*	c.686G>A; c.935dup	Compound heterozygous	p.(Arg229*) (Pathogenic); p.(Ser313Valfs*33) (Pathogenic)	Normal GFR
25a	Female	Yes	3 year	SRNS	FSGS	*NPHS2*	c.779T>A	Homozygous	p.(Val1260Glu) (Pathogenic)	KT
25b	Male	Yes	18 month	SRNS	FSGS	*NPHS2*	c.779T>A	Homozygous	p.(Val1260Glu) (Pathogenic)	KT
26	Female	Yes	3 year	SRNS	FSGS	*NPHS2*	c.779T>A	Homozygous	p.(Val1260Glu) (Pathogenic)	KT
27a	Female	Yes	3 year	SRNS	FSGS	*NPHS2*	c.779T>A	Homozygous	p.(Val1260Glu) (Pathogenic)	KT
27b	Male	Yes	2 year	SRNS	Not done	*NPHS2*	c.779T>A	Homozygous	p.(Val1260Glu) (Pathogenic)	Normal GFR
28a	Male	Yes	4 year	SRNS	FSGS	*NPHS2*	c.467dup	Homozygous	p.(Leu156Phefs*11) (Pathogenic)	Deceased
28b	Male	Yes	2 year	SRNS	Not done	*NPHS2*	c.467dup	Homozygous	p.(Leu156Phefs*11) (Pathogenic)	Normal GFR
29	Female	no	4 year	SRNS	Not done	*NPHS2*	c.779T>A	Homozygous	p.(Val1260Glu) (Pathogenic)	Normal GFR
30a	Male	Yes	1 month	CNS	Not done	*LAMB2*	c.1405+1G>A	Homozygous	Splice donor lost (Pathogenic)	Deceased
30b	Female	Yes	1 month	CNS	Not done	*LAMB2*	c.1405+1G>A	Homozygous	Splice donor lost (Pathogenic)	Deceased
31	Female	No	5 year	SRNS	FSGS	*MYO1E*	c.505C>T	Homozygous	p.(Arg169*) (Pathogenic)	Normal GFR
32a	Male	Yes	7 month	INS	DMS	*PLCE1*	c.4301G>T; c.4306del	Compound heterozygous	p.(Arg1434Leu) (Pathogenic); p.(Val1436*) (Pathogenic)	KT
32b	Male	Yes	2 year	SRNS	DMS	*PLCE1*	c.4301G>T; c.4306del	Compound heterozygous	p.(Arg1434Leu) (Pathogenic); p.(Val1436*) (Pathogenic)	KT
33	Male	Yes	7 year	INS	DMS	*PLCE1*	c.4306del	Homozygous	p.(Val1436*) (Pathogenic)	PD
34a	Female	Yes	4 year	SRNS/CKD	IFTA	*NUP93*	c.1319T>C	Homozygous	p.(Phe440Ser) (Uncertain Significance PP3 PM2 PP1 BP1)	PD
34b	Female	Yes	4 year	SRNS/CKD	IFTA	*NUP93*	c.1319T>C	Homozygous	p.(Phe440Ser) (Uncertain Significance PP3 PM2 PP1 BP1)	PD
35a	Male	Yes	1 month	CNS	Not done	No				Deceased
35b	Male	Yes	2 month	CNS	Not done	No				Deceased
36	Female	No	1 month	CNS	Not done	No				Deceased
37	Female	No	1 month	CNS	Not done	No				Deceased
38	Female	No	3 month	CNS/CKD	Not done	No				PD
39	Female	No	1 month	CNS	Not done	No				CKD
40	Male	No	3 year	SRNS	FSGS	No				Deceased
41	Male	No	4 year	SRNS	FSGS	No				Normal GFR
42	Female	No	4 year	SRNS	FSGS	No				HD
43	Male	No	10 year	SRNS	FSGS	No				KT
44	Male	Yes	10 year	SRNS	FSGS	No				Normal GFR
45	Male	No	2 year	SRN/CKD	FSGS	No				PD
46	Male	No	3 year	SRNS	FSGS	No				Normal GFR
47	Male	No	4 year	SRNS	MCD	No				CKD
48	Female	No	1 year	INS/CKD	FSGS	No				Deceased
49	Female	No	5 month	INS/CKD	FSGS	No				Deceased
50	Female	No	5 year	SRNS	FSGS	No				CKD

*Note*: Reference Sequences: *NPHS1* NM_004646.4; *NPHS2* NM_014625.4; *LAMB2* NM_002292.4; *MYO1E* NM_004998.4; *PLCE1* NM_016341.4; *NUP93* NM_014669.5.

Abbreviations: CKD, chronic kidney disease; CNS, congenital nephrotic syndrome; Comp het, compound heterozygous; DMS, diffuse mesangial sclerosis; FSGS, focal segmental glomerulosclerosis; FT, finish type nephrotic syndrome; HD, hemodialysis; Het, heterozygous; Homo, homozygous; IFTA, interstitial nephritis with tubular atrophy; INS, infantile nephrotic syndrome; KT, kidney transplantation; M, month; MCD, minimal change disease; NGFR, normal glomerular filtration rate; PD, peritoneal dialysis; SRNS, steroid resistance nephrotic syndrome; Y, year. Shading in pale green is for *NPHS1* variants; pale blue for NPHS2 variants; peach for *LAMB2* variants; orange for *MYO1E* variants; green for *PLCE1* variants; blue for *NUP93* variants.

*NPHS2* pathogenic variants were the most common, detected in 36 patients; pathogenic variants in *NPHS1* were detected in 16 patients, pathogenic variants in *PLCE1* were detected in three patients, pathogenic variants in *LAMB2* and *NUP93* were detected in two patients each, and a pathogenic variant in *MYO1E* was detected in one patient (Figure 1b). *NPHS1* was the most frequent genetic cause of CNS whilst *NPHS2* had a range of ages of presentation (Figure 1c).

### 3.2 *NPHS1* genetic findings

Sixteen (21.6%) patients had disease‐causing variants in *NPHS1* and all of them presented with CNS. The mean age at presentation was 1.25 ± 0.57 months (range 0–3 months). Consanguinity was reported in 14 patients and 9 patients had a family history of CNS. Kidney biopsy was performed for 5 patients, all of which showed histopathological features of Finnish‐type NS. Molecular analysis of *NPHS1* showed that 12 patients had homozygous disease‐causing variants, while 4 had compound heterozygous disease‐causing variants. In total, seven different pathogenic variants were detected in *NPHS1* gene (Table 1). The c.515_517 delCCA, p.(Thr172del) was the most common variant, detected in homozygous state in eight patients from five different families. All detected *NPHS1* variants were already known (Heeringa et al., 2008; Schoeb et al., 2010), except for c.2663G>A, p.(Arg888Lys), that was detected in two siblings (family 10) in a homozygous state. Dialysis was performed for six patients, and two of these patients underwent kidney transplantation.

### 3.3 *NPHS2* genetic findings

Thirty‐six patients from 18 different families had disease‐causing variants in *NPHS2*; of these, 28 patients had childhood SRNS and 8 patients had INS, none presented with CNS. The mean age at presentation was 29.8 months. Consanguinity was seen in 34 of these patients and 30 patients had a family history of kidney disease. Kidney biopsy was performed for 21 patients and the histopathologic findings were FSGS in 20 patients and MCD in 1 patient. Thirty‐two of these patients had homozygous causative variant, whereas only four patients had compound heterozygous pathogenic variants. There were six different pathogenic variants detected in *NPHS2* all of which have been already reported previously in HGMD. The most common was c.779T>A, p.(Val1260Glu) seen in homozygous state in 18 patients. Dialysis was performed in 21 patients, and 12 of these patients received a kidney transplant (Table 3).

### 3.4 *LAMB2* , *MYO1E* , *PLCE1* , and *NUP93* genetic findings

Eight patients from four different families were genetically diagnosed using WES. The first family (Family 30) involved two siblings who presented with severe CNS from birth and developed progressive CKD and died within 3 months of age. In addition, both siblings presented with eye phenotypes, including absence of a red reflex, hypopigmented iris with a pinpoint iris. They were found to have a known homozygous splicing variant (c.1405+1G>A) in *LAMB2* that was anticipated to disrupt a highly conserved donor splice site (Bredrup et al., 2008).

A homozygous nonsense variant c.505C>T, p.(Arg169*) in *MYO1E* was detected in a 5‐year‐old female with SRNS (Family 31) with a kidney biopsy demonstrating features of FSGS. The c.505C>T allele has been classified as pathogenic (Feltran et al., 2017; Guaragna et al., 2020). This patient was resistant to prednisolone, mycophenolate and tacrolimus, and immunosuppressive medications were discontinued following the molecular genetic diagnosis, and she was continued on ACE inhibitor that resulted in the preservation of kidney function, with a normal eGFR at the last follow‐up aged 9 years.

**FIGURE 2:**
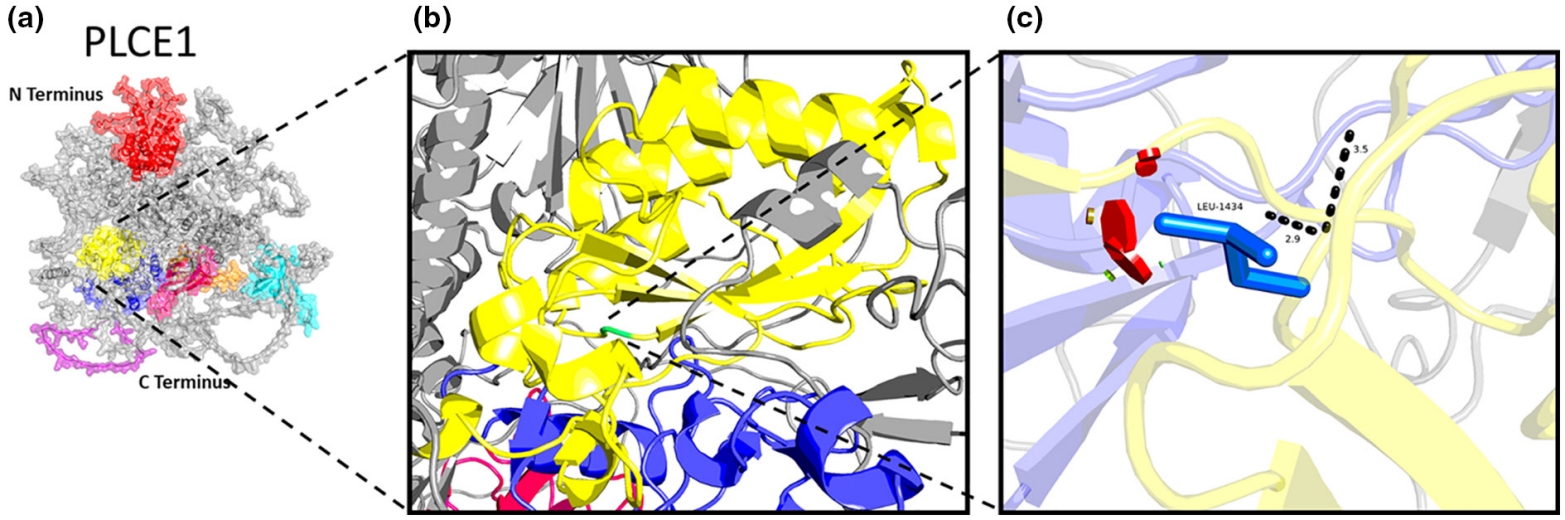
In silico modeling of *PLCE1* p.(Arg1434Leu) missense variant. (a) The PLCE1 protein has several known domains: a 260 amino acid Ras‐GEF domain (p.531–790) (red), 149 amino acid PI‐PLC X‐box domain (p.1392–1540) (yellow), a 117 amino acid PI‐PLC Y‐box domain (p.1730–1846) (dark blue), 101 amino acid C2 domain (p.1856–1956) (pink), 103 amino acid Ras‐associating 1 domain (p.2012–2114) (orange) and a 104 amino acid Ras‐associating 2 domain (p.2135–2238) (cyan). There is a 79 amino acid region (p.1686–1764) that is required for PLCE1 to be activated by RHOA, RHOB, GNA12, GNA13, and G‐beta gamma (purple). (b) The missense p.(Arg1434Leu) variant in the PLCE1 protein (green) occurs in the PI‐PLC X‐box domain (yellow), important for the catalytic function of the protein. (c) Zoom in of the missense p.(Arg1434Leu) variant. The dashed black lines demonstrate the likely loss of the canonical geometry between 2 atoms in this loop with an overlap of each (numerically labeled in Angstroms). The small green disk represents atoms that are almost in contact or slightly overlapping. The red disks represent the significant pairwise overlap of atomic van der Waals radii causing a likely structural ‘clash’ (size of the disk is proportional to the size of the overlap) and the yellow disk lies in between the severity of overlap of red and green. The amino acid volume change is moderate (190.3 to 163.1 Angstroms).

*PLCE1* pathogenic variants were detected in three children from two different families. In two affected siblings (Family 32), who presented with INS and childhood SRNS, compound heterozygous variants (c.4306del, p.(Val1436*); c.4301G>T, p.(Arg1434Leu)) in *PLCE1* gene were detected. The c.4306del, p.(Val1436*) allele is novel and predicted to be disease causing. The c.4301G>T, p.(Arg1434Leu) missense variant is currently classified as a variant of uncertain significance according to the ACMG criteria but has been previously reported in Chinese patients with SRNS (Wang et al., 2017). In silico modeling of this allele demonstrates it is located within the PI‐PLC X‐box domain, important for its catalytic function and that the missense allele is predicted to cause a moderate disruption (Figure 2). Another child (Family 33), presented with INS, and was found to carry the same novel pathogenic allele c.4306del, p.(Val1436*), this time in its homozygous state. A kidney biopsy was performed for these three patients revealing diffuse mesangial sclerosis. All of them developed progressive CKD leading to KF and led to renal replacement therapy in the form of kidney transplantation (Family 32) and dialysis (Family 33).

**FIGURE 3:**
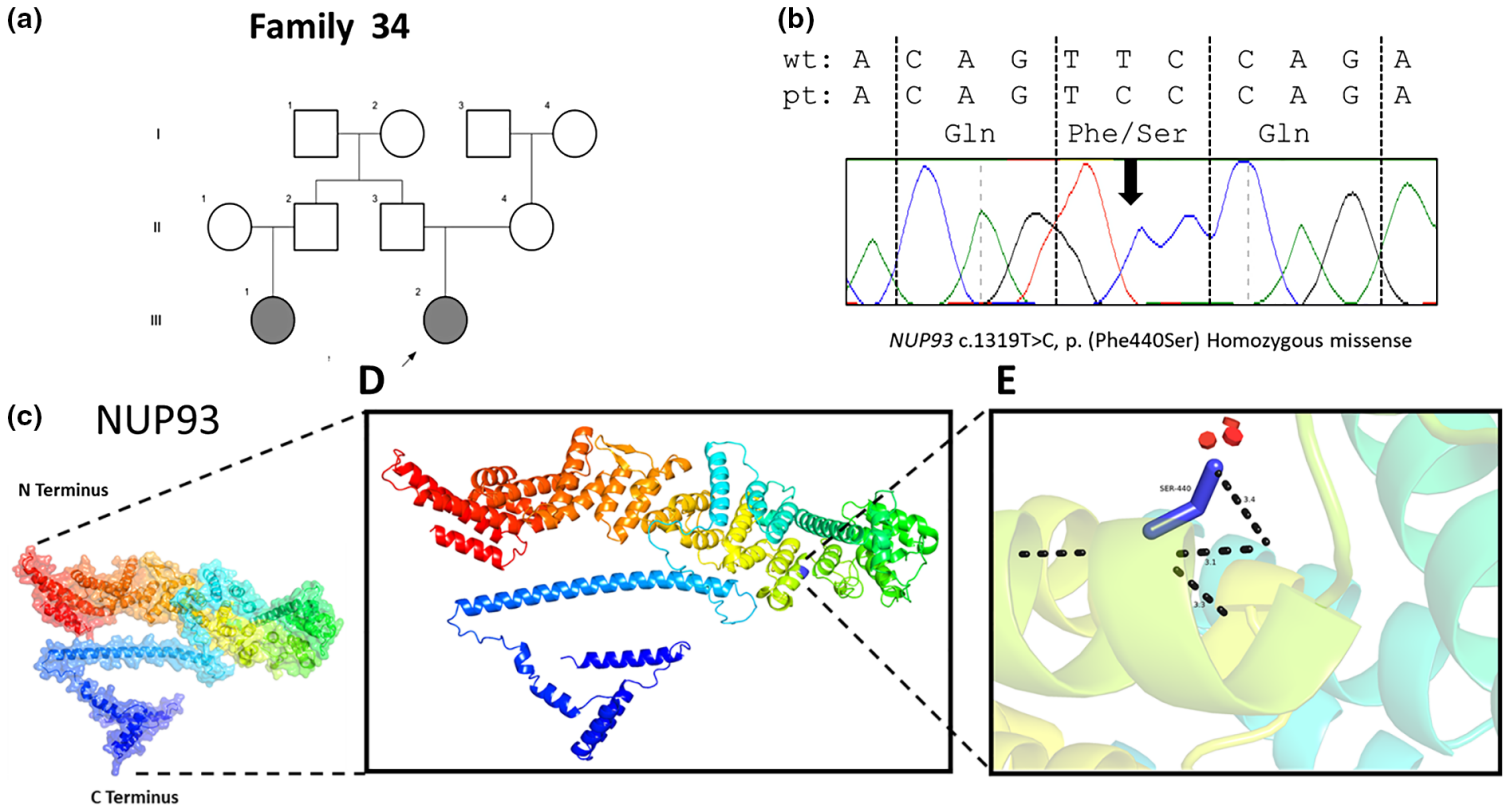
Identification and modeling of a novel *NUP93* missense variant. (a) Pedigree diagram of family 34, with proband and paternal cousin with SRNS diagnosis. (b) Sanger sequencing identified a missense mutation (p.Phe440Ser) in *NUP93* gene (NM_014669.5) in the proband, which was also confirmed in her affected paternal cousin. (c) The predicted three‐dimensional structure of human Nucleoporin 93protein, encoded by *NUP93* on chromosome 16, generated using AlphaFold Protein Structure Database (https://alphafold.ebi.ac.uk) and UniProtKB (https://www.uniprot.org/uniprot/) with associated codes: AF‐Q8N1F7‐F1 and Q8N1F7 respectively. (d) Region (labeled in blue) in which the c1319T>C p.(Phe440Ser) lies in an alpha helix near the middle of the protein. (e) Missense SNV c1319T>C, p.(Phe440Ser) modeled with the most probable (41.9% accuracy) rotamer demonstrated. The dashed black lines demonstrate the likely loss of the canonical geometry between two atoms in this loop with an overlap of each (numerically labeled in Angstroms). The red disks represent the significant pairwise overlap of atomic van der Waals radii causing a likely structural ‘clash’ (size of the disk is proportional to the size of the overlap). The amino acid volume change is very significant (190.8 to 93.5 angstroms) suggesting a significant impact of the mutation on the side‐chain structure.

Family 34 included an affected female and her paternal cousin, both presenting with hypertension, iron deficiency anemia, and reaching KF. WES identified a novel homozygous missense allele c.1319T>C, p.(Phe440Ser) in *NUP93* (NM_014669.5). Pathogenic variants in *NUP93* are associated with NS, type 12 (OMIM 614351) (Braun et al., 2016). Both homozygous missense and compound heterozygous truncating variants with missense variants have been reported in *NUP93* in individuals with SRNS (Braun et al., 2016). The c.1319T>C variant has not previously reported in any databases and in silico analysis including structural modeling suggested it is pathogenic, affecting a highly conserved amino acid residue. Segregation of this causative allele within family members was confirmed (Figure 3) adding weight to its disease causality.

### 3.5 Children without a genetic diagnosis

We were unable to genetically diagnose 17 patients (mean age at presentation 31.7 months) using Sanger sequencing screening of *NPHS1* and *NPHS2* (*n =* 15) and WES (*n =* 2). Six of these patients presented with CNS, nine had childhood SRNS, and the remaining two presented with INS. Nine of them have CKD at presentation. A kidney biopsy was performed in nine patients, of which eight had FSGS and one had MCD (Table 1, Figure 1a). KF requiring dialysis was performed in 13 patients, and one patient received a kidney transplant. Eight patients died (Table 3); of those five had CNS, two had INS, and one had childhood SRNS.

### 3.6 Genotype/phenotype correlations

In this study, patients with SRNS in whom we detected disease‐causing genetic variants were slightly younger (median age ~ 25 months) compared with children without genetic causes (median age 32 months). The median age for those with CNS and diagnosed with *NPHS1* was 1.25 months (Table 2). Consanguinity was common within this study cohort, and a family history of NS was more commonly seen in children with a genetic cause (*NPHS1*: 56%, *NPHS2*: 81.1% and other genes: 87.3%) compared with children without an identified disease‐causing genetic variant (12.5%). CKD at presentation was more common in patients who had an identified genetic cause in *LAMB2*, *PLCE1*, *MYO1E*, and *NUP93* and in those without mutation compared with those with *NPHS1* or *NPHS2* disease‐causing variants.

**TABLE 2:** Clinical features of studied patients with SRNS.

	Total	*NPHS1*	*NPHS2*	Other genes	No pathogenic variant defined
Number of patients	77	16	36	8	17
Gender (Female/Male)	41/36	11/5	18/18	5/3	8/9
Mean age (month)		1.25	29.83	24.6	31.7
Consanguinity	58	14	34	7	3
Family history of nephrotic syndrome	48	9	30	7	2
Hematuria	60	9	32	7	12
CKD at presentation	23	2	5	7	9
Hypothyroidism	47	15	18	6	8
Hypertension at presentation	21	0	9	6	6
Mean eGFR at presentation (mL/min/1.73 m^2^)	124	87	165	18	85
Mean eGFR last follow‐up (mL/min/1.73 m^2^)	53	40	53	12	76
Mean albumin (g/L) at presentation	12.3	7.7	12.5	7.0	16.3
Mean albumin (g/L) last follow‐up	26.0	21.3	28.7	12.5	26.7

Abbreviations: CKD, chronic kidney disease; eGFR, estimated glomerular filtration rate.

### 3.7 Modality of treatment, follow‐up, and outcomes

The 24 patients presenting with CNS were not given immunosuppression treatment, including all of the patients with *NPHS1* mutations. Of the remaining 53 patients, 25 patients received immunosuppressive medication (including steroids with or without other immunosuppressive medication; ciclosporin, tacrolimus, mycophenolate, or rituximab) during the mean follow‐up period of 59.3 months, (Table 3). Of those 25, 16 patients had *NPHS2* variants, 2 had genetic diagnosis in other genes and 7 were without a genetic cause detected. Aside from a CNS presentation other reasons for withholding / avoiding immunosuppression included a known family history of *NPHS2* mutation (11 cases) and KF at presentation. KF requiring dialysis was necessary for 45 patients, while kidney transplantation was performed for 17 patients. During follow‐up period, 21 patients died, 7 of them had *NPHS1* disease‐causing variants, 4 had *NPHS2* disease‐causing mutation, 2 had *LAMB2* gene disease‐causing variants, and 8 had no identified disease‐causing variants detected (Table 3).

**TABLE 3:** Summary of the clinical follow‐up, treatment, and outcome of the studied cohort.

	Total	*NPHS1*	*NPHS2*	Other genetic causes	No identified genetic cause
Mean follow‐up period (month)	59.3	53.1	74.9	31.1	39.4
Immunosuppressive medications	25	0	16	2	7
KF requiring dialysis	45	6	21	5	13
Kidney transplantation	17	2	12	2	1
Death	21	7	4	2	8

## 4 DISCUSSION

This study is the first to describe clinical features, genetic analysis, and outcomes of Omani children with SRNS. It comprised 77 children from 50 different families who presented over a 15‐year period. In this study, 60 out of 77 patients had a single gene defect, whereas 17 remained genetically undiagnosed. However, 15 of the undiagnosed cases were only screened for *NPHS1* and *NPHS2* using direct Sanger sequencing, meaning that an alternative molecular genetic diagnosis may have been missed in these cases. Just 2 cases remained undiagnosed following both targeted Sanger sequencing followed by WES approaches.

*NPHS1* and *NPHS2* were the two major genetic causes identified in this cohort of SRNS children from Omani. Other genetic causes identified were *LAMB2*, *PLCE1*, *NUP93*, and *MYOE1*. These data may be compared with other reports of SRNS patients. In Saudi Arabia, the most commonly identified genes causing SRNS were *NPHS2* (22%), *NPHS1* (12%), *PLCE1* (8%), and *MYOE1*(6%) (Al‐Hamed et al., 2013). In a Japanese SRNS population, the most common genetic causes were seen in *WT1* (25%), *NPHS1* (12%), *INF2* (12%), *TRPC6* (10%), and *LAMB2* (9%) (Nagano et al., 2020), while in China the genetic causes were *ADCK4* (6.67%), *NPHS1* (5.83%), *WT1* (5.83%), and *NPHS2* (3.33%) (Wang et al., 2017). European studies demonstrate a comparable spread of genetic variants in SRNS patients with the most common causes being *NPHS1* (17%), *WT1* (14%), and *NPHS2* (11%) (Morello et al., 2020).

Our study revealed a high rate of molecular genetic diagnoses of SRNS, which is consistent with previous reports (Al‐Hamed et al., 2013; Santín et al., 2011). Most of our patients with genetic causes were born to consanguineous parents and consistent with this, most of the disease‐causing variants were detected in their homozygous state.

In our cohort, disease‐causing variants in *NPHS2* were the most common causes of SRNS accounting for 47%, higher than the rate reported in Europe (40%) (Sadowski et al., 2015) and Turkey (29.9%) (Berdeli et al., 2007). We also observed that *NPHS2* disease‐causing variants were detected in infants and children ranging between 5 months and 6 years at disease presentation, but were not identified in any patients presenting with CNS. In comparison, previous studies of CNS in non‐Finnish patients reported both *NPHS1* and *NPHS2* disease‐causing variants, contributing up to 75% of the genetic causes, but with patients with *NPHS2* being less likely to present before first month of life (Machuca et al., 2010; Sadowski et al., 2015).

The most common disease‐causing *NPHS2* variant in this study was the homozygous missense allele c.779T>A, p.(Val1260Glu), which was found in 18 of our patients. This allele was previously reported in Saudi Arabian patients (Al‐Hamed et al., 2013) and in four patients from Europe and North Africa by Weber et al. (2004). The second most common *NPHS2* allele, c.467dup, p.(Leu156Phefs*11) was seen in 11 patients.

In our study, disease‐causing mutations have been identified in 75% of our CNS cohort, where *NPHS1* was the most common causative gene. Our findings are consistent with the reported diagnostic rate of 75%–100% of cases of CNS (Preston et al., 2019). Most of our patients with *NPHS1* variants had a homozygous disease‐causing variant, whereas 4 had compound heterozygous alleles. None of the Fin _major_ or Fin _minor_ variants were detected in our cohort, which was consistent with a previous study that showed the rarity of these mutations in non‐Finnish patients (Al‐Hamed et al., 2013; Beltcheva et al., 2001; Heeringa et al., 2008). Other studies in non‐Finnish patients found only two patients had Fin _major_ or Fin _minor_ mutations (Machuca et al., 2009). A kidney biopsy was performed for five CNS patients and all showed features of Finnish‐type NS. Most of our patients presented within 1 month of life and progressed to KF at the mean age of 31 months.

A homozygous pathogenic variant in *LAMB2* gene was identified in 2 siblings (Family 30) who presented with CNS confirming the diagnosis of autosomal recessive NS type 5, this variant was previously reported by Bredrup et al. (2008) and Matejas et al. (2010). These 2 children progressed rapidly to KF and died within 3 months, along with minor eye abnormalities, consistent with a unifying diagnosis of Pierson syndrome. Pierson syndrome (OMIM 609049) is an autosomal recessive disorder secondary to variants in *LAMB2* and is characterized by CNS, neurodevelopment abnormalities and eyes anomalies including microcoria and hypoplasia of the ciliary and pupillary muscles (Zenker et al., 2004). Usually, these symptoms present in utero or within the first 3 months of life, as in our identified patients. In contrast, Kagan et al. (2008) reported a mild presentation of an infant with CNS, who had minor eye abnormalities without any neurological abnormalities and developed KF at 16 months of age.

A novel *PLCE1*variant was identified in our cohort (Family 32 and 33) that is a frameshift deletion variant c.4306del, p.(Val1436*). All patients with *PLCE1* pathogenic variants developed progressive CKD and started renal replacement therapy (dialysis/kidney transplantation). Pathogenic variants in *PLCE1* gene are associated with NS type 3 and patients typically have early onset and severe NS with rapid progression to CKD, where most of them illustrate DMS and some FSGS on kidney biopsy (Hinkes et al., 2006). Although previous reports showed a partial response of NS patient with disease‐causing mutations in *PLCE1* to tacrolimus therapy, further studies are still needed to determine the reno‐protective effect of such proteinuria reduction (Lin et al., 2014).

In this study, we identified only one child with a homozygous nonsense variant in *MYO1E* which was reported previously (Feltran et al., 2017; Guaragna et al., 2020). This patient's NS was resistant to immunosuppressant medications. *MYO1E* gene encodes myosin 1E, which is a non‐muscle class 1 myosin, that regulates the podocyte cytoskeleton (Kaplan et al., 2000; Kim et al., 2003). The *MYO1E* variants are associated with childhood onset autosomal recessive FSGS (Mele et al., 2011). The *MYO1E* is uncommon etiology for childhood FSGS but is seen more frequently in consanguineous families (Al‐Hamed et al., 2013; Mele et al., 2011).

The genetic findings of our study have a valuable impact on the early clinical management of studied children as well as future genetic counseling for affected families. The detection of causative variants in NS‐associated genes clarifies the accurate mode of inheritance and facilitates proper counseling of family members as well as guides the setting for kidney transplant and selection of living related kidney donors. Our study has some limitations including the small cohort size as well as the genetic screening methodologies used. Initially, we focused on the targeted screening of two main genes *NPHS1* and *NPHS2* and only selective cases were taken forward for WES, due to local resource limitations. To improve detection rates, searching for disease‐causing variants in all monogenic NS‐causing genes is required through modern NGS approaches including targeted gene panels, WES and whole‐genome sequencing. Such strategies of molecular genetic diagnosis are anticipated to improve our understanding of etiology of SRNS in distinct populations and enhance personalized medicine in the term of individualizing management and avoiding immunosuppressive medications.

## 5 CONCLUSION

*NPHS1* and *NPHS2* disease‐causative variants were the most commonly detected molecular genetic causes of SRNS in Omani children. Using WES increases the detection ability of the other rarer genetic causes of SRNS. In view of this, NGS screening of all known genes underlying NS should be implemented in all children who present with SRNS to allow optimization of treatment and prediction of kidney survival outcomes.

## AUTHOR CONTRIBUTIONS

MSAR and IAA conceived the study and wrote the first draft of the manuscript. Clinical and molecular data analysis was performed by MSAR, IAA, BAG, AAM, NAK, NAAH, AAB, MAS, SAS, MAB, and FAH. In silico modeling was performed by HM. JAS coordinated the work and edited the manuscript. All authors read and approved the final manuscript.

## CONFLICT OF INTEREST STATEMENT

The authors declare no conflicts of interest.

## ETHICS STATEMENT

This study was ethically approved by the Royal Hospital Research Ethical Committee, Ministry of Health (MOH/CSR/21/24412). For genetic studies of patients, written informed consent was provided by the patients' families.

## Data Availability

The data are not available for public access because of patient privacy concerns but are available from the corresponding author on reasonable request.
